# Supplementary figures and images for: Synergistic effects of Rhynchosia nulubilis and Polygonum multiflorum extract combination on cell proliferation via targeting IGFBP-1 & NT-3 and cytotoxicity suppression in testosterone-induced human dermal papilla cells
Source: PLoS One. 2025 May 27;20(5):e0321812. doi: 10.1371/journal.pone.0321812 (PMC12111526; doi:10.1371/journal.pone.0321812)

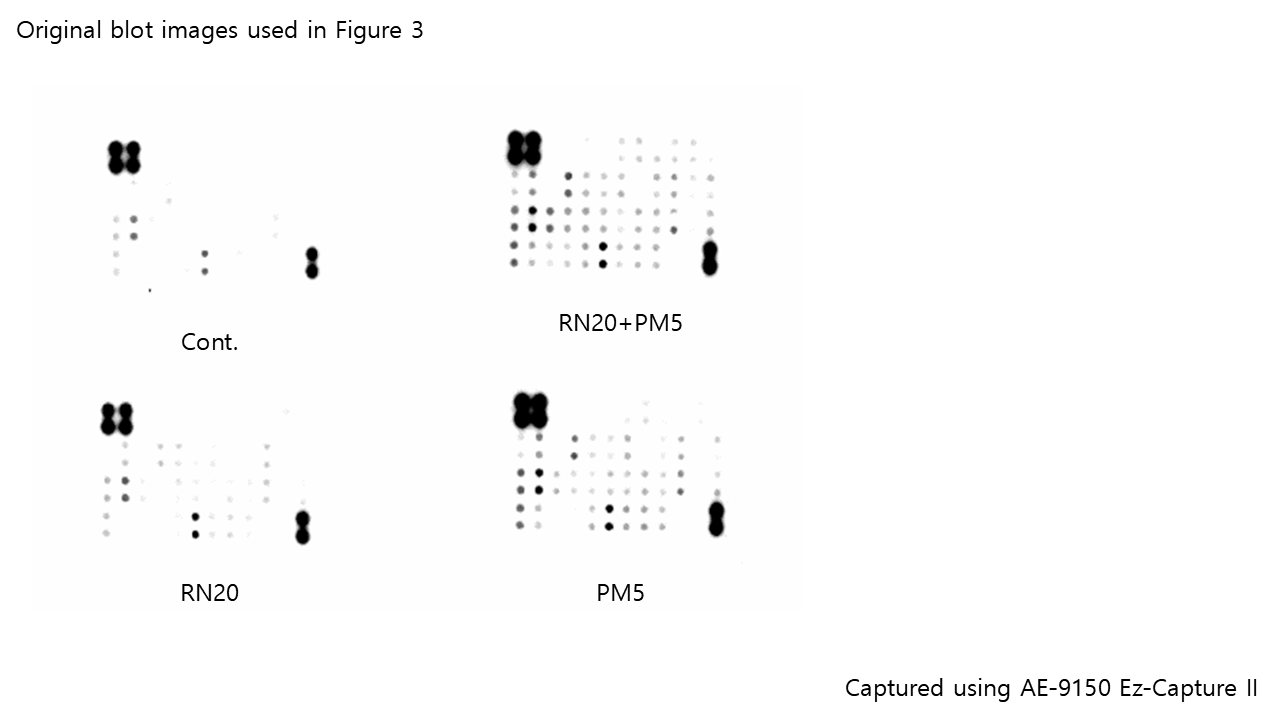

Supplement: S1 File — S1 Data. (1–3) Raw growth factor array, western blot image. S2 Data. Raw data in the manuscript. (ZIP) [file pone.0321812.s001.zip › Supporting Information/S1-1.Raw data.TIF]

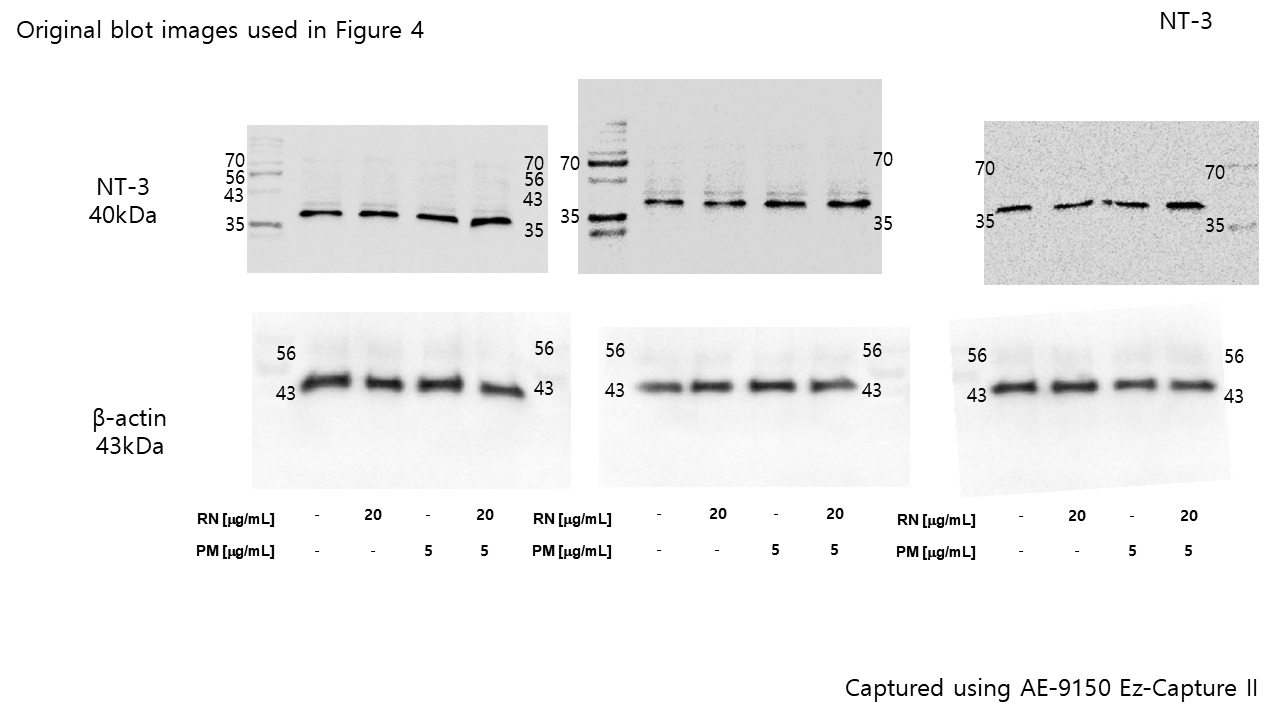

Supplement: S1 File — S1 Data. (1–3) Raw growth factor array, western blot image. S2 Data. Raw data in the manuscript. (ZIP) [file pone.0321812.s001.zip › Supporting Information/S1-2.Raw data.TIF]

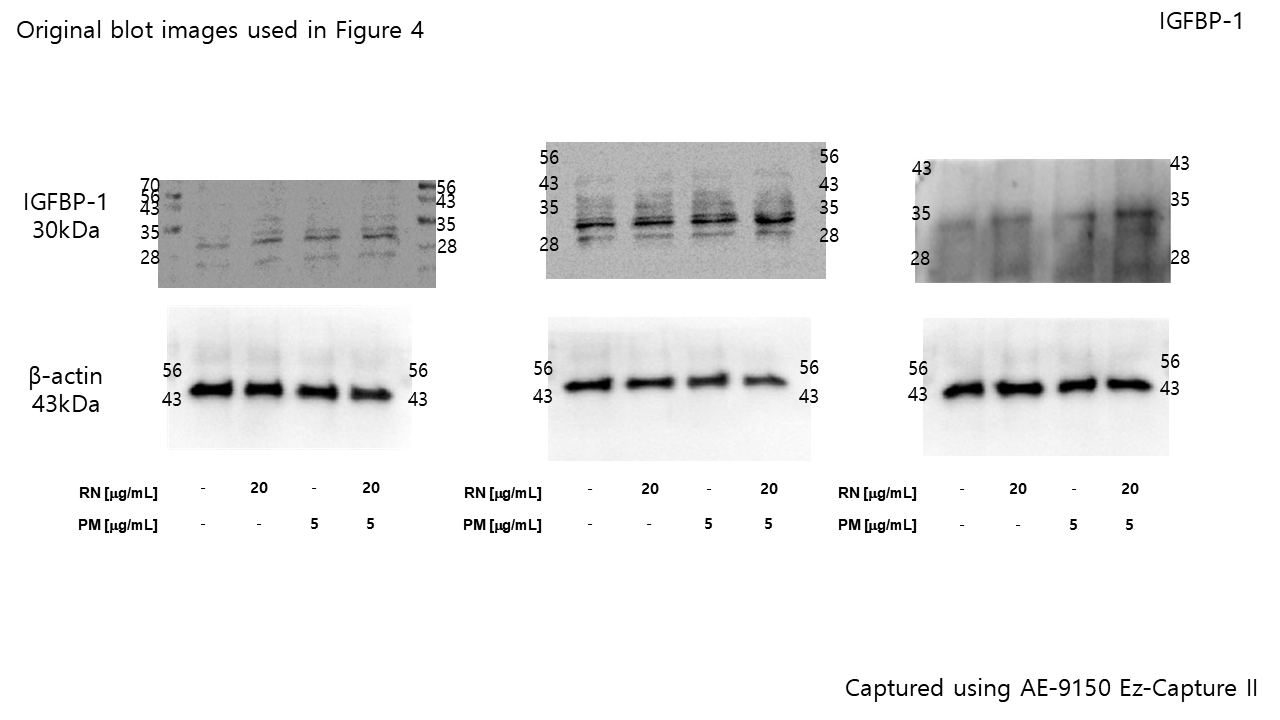

Supplement: S1 File — S1 Data. (1–3) Raw growth factor array, western blot image. S2 Data. Raw data in the manuscript. (ZIP) [file pone.0321812.s001.zip › Supporting Information/S1-3.Raw data.TIF]
